# A deimmunised form of the ribotoxin, α-sarcin, lacking CD4^+^ T cell epitopes and its use as an immunotoxin warhead

**DOI:** 10.1093/protein/gzw045

**Published:** 2016-10-22

**Authors:** Tim D. Jones, Arron R. Hearn, Robert G.E. Holgate, Dorota Kozub, Mark H. Fogg, Francis J. Carr, Matthew P. Baker, Javier Lacadena, Kurt R. Gehlsen

**Affiliations:** 1 Abzena plc., Babraham Research Campus, Babraham, CambridgeCB22 3AT, UK; 2 Abtelum Biomedical, Inc. 175 Briar Lane, Westwood, MA 02090, USA; 3 Departamento de Bioquimica y Biologia Molecular I, Facultad de Ciencias Químicas, Universidad Complutense de Madrid, Avenida Complutense s/n, Madrid 28040, Spain; 4 Research Corporation Technologies Inc., 5210 E. Williams Circle #240, Tucson, AZ 85711, USA

**Keywords:** α-sarcin, deimmunisation, immunogenicity, immunotoxin, T cell epitope

## Abstract

Fungal ribotoxins that block protein synthesis can be useful warheads in the context of a targeted immunotoxin. α-Sarcin is a small (17 kDa) fungal ribonuclease produced by *Aspergillus giganteus* that functions by catalytically cleaving a single phosphodiester bond in the sarcin–ricin loop of the large ribosomal subunit, thus making the ribosome unrecognisable to elongation factors and leading to inhibition of protein synthesis. Peptide mapping using an *ex vivo* human T cell assay determined that α-sarcin contained two T cell epitopes; one in the N-terminal 20 amino acids and the other in the C-terminal 20 amino acids. Various mutations were tested individually within each epitope and then in combination to isolate deimmunised α-sarcin variants that had the desired properties of silencing T cell epitopes and retention of the ability to inhibit protein synthesis (equivalent to wild-type, WT α-sarcin). A deimmunised variant (D9T/Q142T) demonstrated a complete lack of T cell activation in *in vitro* whole protein human T cell assays using peripheral blood mononuclear cells from donors with diverse HLA allotypes. Generation of an immunotoxin by fusion of the D9T/Q142T variant to a single-chain Fv targeting Her2 demonstrated potent cell killing equivalent to a fusion protein comprising the WT α-sarcin. These results represent the first fungal ribotoxin to be deimmunised with the potential to construct a new generation of deimmunised immunotoxin therapeutics.

## Introduction

Immunotoxins are chimeric molecules containing a targeting moiety linked to a toxin (Alewine *et al*., 2015; Wayne *et al*., 2014) and are primarily used as therapeutics to specifically target cells for destruction by the toxin while avoiding normal healthy cells. The targeting portion can be an antibody, antibody fragment or some other targeting protein (e.g. abdurin, cytokine, peptide, ligand and hormone) designed to bind to a specific cell-surface molecule or receptor. Ontak^TM^ is the first, and only, immunotoxin approved by the FDA for the treatment of certain B cell leukaemias (Wayne *et al*., 2014). It comprises an interleukin-2 receptor targeting moiety, in the form of interleukin-2 fused to a truncated form of diphtheria toxin, which is a very potent inhibitor of protein synthesis.

Many toxin warheads have been investigated for potential development as immunotoxin-based therapeutics including: ricin, diphtheria toxin, pseudomonas exotoxin A, gelonin, bouganin, saponin and various mutants of each (Alewine *et al*., 2015; Antignani and Fitzgerald, 2013; Carreras-Sangrà *et al*., 2008). A number of these immunotoxins have been tested in clinical trials, particularly PE38 (a truncated pseudomonas exotoxin A variant), gelonin and ricin (Borthakur *et al*., 2013; Hassan *et al*., 2013, 2014; Kreitman *et al*., 2000, 2009; Schnell *et al*., 2003; Schindler *et al*., 2011). Unfortunately, all of these trials have reported the development of treatment-limiting anti-drug antibodies (ADA), although the frequency of patients developing ADA (varying from a few per cent to 90%) depended upon the disease and their immune competence. Furthermore, the presence of pre-existing ADA as a result of vaccination or environmental exposure can also interfere with treatment (Hu, 1995; Matar *et al*., 2012; Manning *et al*., 2015). The immunogenicity of toxins that are derived from plants or microbial sources almost certainly arises as a result of a lack of immunological tolerance, i.e. the non-human protein is recognised as foreign by the patient's immune system. It is possible to address immunogenicity that arises as a consequence of the ‘foreignness’ of a protein by mutating B and/or T cell epitopes. The limitation of targeting B cell epitopes recognised by the pre-existing ADA (Nagata and Pastan, 2009) is that the ADA response can develop upon repeated treatment with the protein resulting in the recognition of new epitopes through a process of epitope spreading and this process is driven by the presence of CD4^+^ T cell epitopes in the protein sequence. It is known that CD4^+^ T cell epitopes are the principle drivers of ADA responses *in vivo* (Baker and Carr, 2010). Eliminating T cell epitopes will, therefore, prevent the generation of both T cell and B cell responses, an approach that has recently been applied to PE38 (Mazor *et al*., 2014).

Ribotoxins constitute a family of cytotoxic secreted fungal RNases, best represented by α-sarcin (Lacadena *et al*., 2007). They cleave a single phosphodiester bond of the larger rRNA, located at the universally conserved site in the sarcin–ricin loop (SRL). Cleavage of the SRL results in ribosome inactivation leading to protein biosynthesis inhibition and cell death (García-Ortega *et al*., 2010; Lacadena *et al*., 2007; Martínez-Ruiz *et al*., 2001; Olombrada *et al*., 2014). Fungal ribotoxins have several advantages in the context of developing a therapeutic immunotoxin, these include small size, high thermostability, resistance to proteases and their highly efficient ability to inactivate ribosomes (Goyal and Batra, 2000; Lacadena *et al*., 2007; Rathore *et al*., 1997). They have not, however, been so frequently employed as bacterial toxins in the development of therapeutic immunotoxins, and so far only preliminary characterisation has been reported (Better *et al*., 1992; Goyal and Batra, 2000; Rathore *et al*., 1997; Wawrzynczak *et al*., 1991). Most recently, a colon cancer-specific immunotoxin based on α-sarcin has been described (Carreras-Sangrà *et al*., 2008) that exhibited highly specific antitumour activity in mice harbouring colon tumour xenografts (Tomé-Amat *et al*., 2015b). These results support the potential clinical application of ribotoxin-based immunotoxins, and particularly those based on α-sarcin. Although low immunogenicity has been reported for certain ribotoxins (Goyal and Batra, 2000; Rathore and Batra, 1996), given the non-human source of the protein there is still a significant risk of immunogenicity which must be considered before development as a therapeutic immunotoxin for use in humans. Thus, it is essential for long-term treatment of patients to have non-immunogenic toxins.

To this end, we sought to deimmunise α-sarcin using a preclinical *ex vivo* human T cell assay (EpiScreen^TM^) (Jones *et al*., 2004) to identify linear T cell epitopes present within the α-sarcin sequence. Overlapping peptides spanning the entire sequence were tested for the capacity to induce T cell proliferation against a cohort of community blood donors carefully selected based on expression of a broad range of major histocompatibility complex (MHC) class II haplotypes. Using this assay, two T cell epitopes were identified within α-sarcin: Epitope 1 (Residues 10–18) and Epitope 2 (Residues 134–142). Guided mutational design was used to predict changes that could be made within the identified T cell epitopes that eliminated MHC class II binding and therefore removed the epitopes. Selection of deimmunised α-sarcin variants was based on retaining equal or better cytotoxicity as wild-type (WT) α-sarcin in *in vitro* transcription and translation (IVTT) and *in vitro* cellular toxicity assays; as well as deimmunised α-sarcin being non-immunogenic (compared to WT α-sarcin) when tested as a whole protein in the *ex vivo* T cell assay (Holgate *et al*., 2015). A deimmunised α-sarcin variant containing the mutations D9T and Q142T was as potent as WT α-sarcin and demonstrated complete silencing of the T cell epitopes when tested in the *ex vivo* T cell assay.

## Materials and methods

### *In Vitro* Transcription/Translation assay

IVTT assays were performed using DNA directly in the assay; the gene encoding WT α-sarcin corresponding to Residues 1–150 (Genbank accession no. BAA02863, amino acids 28–177) and containing a C-terminal stop codon was cloned into the T7 expression plasmid pET22b (Millipore UK Ltd, Watford, UK) downstream of the Nde I site. In addition, a null mutant of WT α-sarcin with the mutation, H137Q (α-sarcin-H137Q) (Lacadena *et al*., 1999), was generated as a negative control using PCR-based site directed mutagenesis. Plasmid DNA was purified using a Qiaprep Spin Mini-prep Kit (Qiagen, Manchester, UK). The cell-free IVTT assay was performed with a TnT^®^ T7 Coupled Reticulocyte Lysate System (Promega, Southampton, UK) according to the manufacturer's instructions, with some modifications. pET22b plasmids containing either WT α-sarcin or α-sarcin-H137Q were tested at concentrations ranging from 400 to 6.25 ng per 12.5 µl reaction. The test DNA was combined with the IVTT reaction mix supplemented with 0.1% Triton X-100, 0.2 mM MgCl_2_ and 12.2 mM KCl and incubated at 22°C for 45 min. Following a subsequent cooling step at 4°C for 5 min, 250 ng of T7 luciferase plasmid provided with the TnT^®^ T7 Coupled Reticulocyte Lysate System were added and the reactions incubated at 24°C for a further 90 min. The reactions were stopped by placing the samples on ice and luciferase activity was measured using Steady Glo reagent (Promega, Southampton, UK), according to the manufacturer's instructions. Luminescence was measured in a FluoStar Optima plate reader (BMG Labtech, Ortenberg, Germany).

### Protein expression

Trastuzumab (anti-Her2) scFv-sarcin fusions were cloned into pET22b downstream of a modified outer membrane protein A leader peptide, which has been shown to have improved processing and export compared to the original sequence (Lacadena *et al*., 1994), and upstream of a 6× His tag. Initially, a furin cleavage site (RSKR) (Hosaka *et al*., 1991) was engineered between the scFv and the α-sarcin sequence to enable cleavage and release of mature toxin following internalisation and transport to the Golgi in target cells (Goyal and Batra, 2000); later constructs contained a G_4_S linker. Two null mutants of α-sarcin were also generated in the same configuration to serve as negative controls, namely α-sarcin-H137Q and α-sarcin-E96Q (Lacadena *et al*., 1999). In order to express the α-sarcin variants (including null-mutants H137Q and E96Q), an *Escherichia coli* BL21-DE3 strain Shuffle™ T7 Express (NEB, Hitchin, UK) derivative overexpressing the chaperonins GroEL/S was used. Bacteria were transformed with expression plasmids and plated out. Single colonies were picked and grown in 2YT broth overnight at 37°C. The following day, the overnight culture was diluted 1:20 in 2YT broth and bacterial growth at 37°C was monitored by OD_600_ measurement. Protein expression was induced at OD_600nm_ = 1.0 by adding IPTG to give a final concentration of 1 mM and the culture was then grown at 20°C overnight before cells were harvested by centrifugation and frozen overnight at −80°C. Cell pellets were resuspended in B-PER (ThermoFisher, Waltham, MA) containing DNase I (Roche Diagnostics Ltd, Burgess Hill, UK) and protease inhibitors (Roche Diagnostics Ltd, Burgess Hill, UK). The insoluble protein was removed by centrifugation at 26 000 *g* for 30 min. Soluble protein was diluted 2-fold in 40 mM Tris-HCl pH 7.5, 300 mM NaCl, 80 mM imidazole and clarified by centrifugation before addition of 1 ml Ni-NTA-agarose (Qiagen, Manchester, UK) pre-equilibrated with 20 mM Tris-HCl pH 7.5, 300 mM NaCl and 40 mM imidazole (binding buffer). After incubation with rotation overnight at 4°C, unbound protein was removed by centrifugation of the Ni-NTA-agarose followed by a 10-column volumes wash with binding buffer. A stepwise wash/elution was then performed starting with a 10-column volumes wash with 20 mM Tris-HCl pH 7.5, 300 mM NaCl, 100 mM imidazole (wash buffer) followed by elution with 20 mM Tris-HCl pH 7.5, 300 mM NaCl, 400 mM imidazole (elution buffer). One millilitre elution fractions were collected and analysed by SDS-PAGE. Fractions containing the protein of interest were pooled, buffer exchanged into PBS pH 7.4 and soluble protein quantified by OD_280nm_ (*E*c(0.1%) = 1.57).

### Cellular cytotoxicity assays

For anti-Her2 scFv-sarcin fusions, the Her2 + human breast cancer cell line BT-474 (LGC Standards, Teddington, UK) was used as the target. Briefly, cells in the log phase of growth were harvested, diluted to 4 × 10^4^ cells/ml in normal growth media DMEM/F-12 (Life Technologies, Paisley, UK) and 100 μl dispensed into each well of a 96-well white-walled tissue culture plate (Greiner Bio-One, Stonehouse, UK). Outer wells contained growth media only in order to avoid edge effects. Cells were incubated overnight in a humidified cell culture incubator (37°C, 5% CO_2_). A dilution plate was prepared containing a 3-fold dilution (from either 702–0.04 or 234–0.01 nM) in normal growth media across the plate of each test sample, and 50 µl of each dilution series was transferred directly onto the BT-474 cells, from which 50 µl of media had been removed, before returning the cells to the incubator and incubations were carried out for 7 days. After incubation, the plate was equilibrated at room temperature for 10 min and then developed by the addition of 100 µl of Cell TiterGlo reagent (Promega, Southampton, UK) to each well prior to reading in a FluoStar Optima plate reader (BMG Labtech, Aylesbury, UK).

### *Ex vivo* T cell assay donor selection

Peripheral blood mononuclear cells (PBMCs) were isolated from healthy community donor buffy coats (from blood drawn within 24 h) with appropriate consent and obtained from the UK National Blood Transfusion Service (Addenbrooke's Hospital, Cambridge, UK). PBMCs were isolated from buffy coats by Lymphoprep (Axis-shield, Dundee, UK) density centrifugation and CD8^+^ T cells were depleted using CD8^+^ RosetteSep™ (StemCell Technologies Inc, London, UK). Human leukocyte antigen-D related (HLA-DR) haplotypes were determined using an HLA sequence specific primer- PCR based tissue-typing kit (Biotest, Solihull, UK). PBMCs were frozen and stored in liquid nitrogen until required.

### *Ex vivo* human T cell assays

#### T cell epitope mapping

Fifteen-mer peptides overlapping by 12 amino acids spanning the entire WT α-sarcin sequence, plus peptides spanning the null mutations E96Q and H137Q, were synthesised on a 1–3 mg scale with free N-terminal amine and C-terminal carboxylic acid (Mimotopes, Clayton, Australia). Peptides were dissolved in DMSO to a concentration of 10 mM and peptide culture stocks prepared by diluting into AIM-V^®^ culture medium (Gibco, Paisley, UK) to a final concentration of 5 µM. Peptide assays were performed as previously described (Jones *et al*., 2004) using a cohort of 52 donor PBMC. Briefly, for each peptide and each donor, sextuplicate cultures were established in a flat bottomed 96-well plate. For a positive control, phytohaemagglutinin (PHA, Sigma, Poole, UK) was used at a final concentration of 2.5 µg/ml and also tested in sextuplicate. Cultures were incubated for a total of 6 days before adding 0.75 µCi ^3^[H]-thymidine (Perkin Elmer^®^, Beaconsfield, UK) to each well. Cultures were incubated for a further 18 h before harvesting onto filter mats using a TomTec Mach III cell harvester. Counts per minute (CPM) for each well were determined by Meltilex™ (Perkin Elmer^®^, Beaconsfield, UK) scintillation counting on a Microplate Beta Counter (Perkin Elmer^®^, Beaconsfield, UK in paralux, low background counting mode).

Positive responses were defined by a stimulation index (SI = mean cpm of test wells /mean cpm medium control wells) equal to or greater than 1.9 (SI ≥ 1.90) where the average cpm in the test wells was significantly (*P *< 0.05) different to the average cpm of the medium control wells using an unpaired two sample Student's *t*-test. T cell epitopes were identified by calculating the average frequency of positive responses (defined above) to all peptides in the study plus standard deviation to give a background response rate. Any peptide that induced proliferative responses above the background response rate was considered to contain a T cell epitope.

#### Whole protein assays

**Fig. 1:**
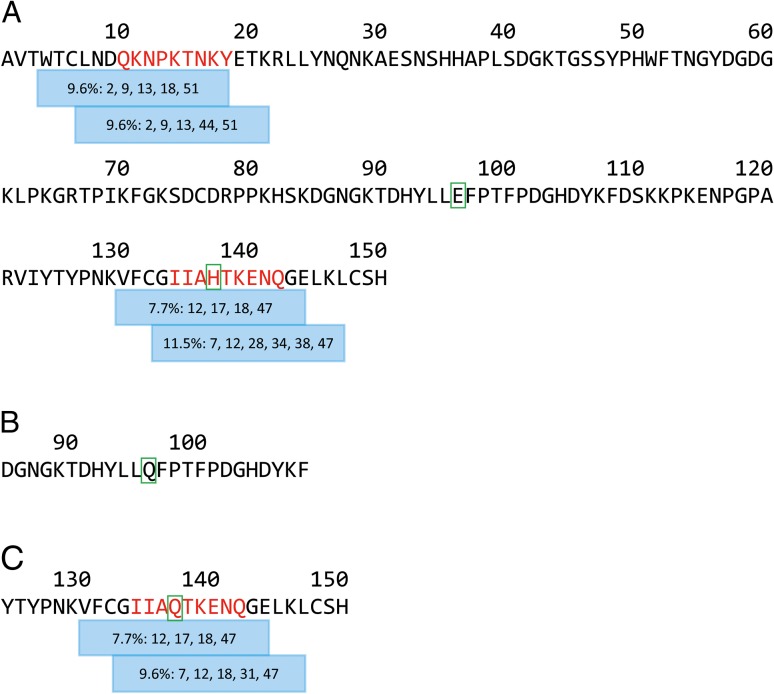
CD4^+^ T cell epitope map of the (**A**) α-sarcin toxin, (**B**) null-mutant E96Q and (**C**) null-mutant H137Q sequences. Peptides containing T cell epitopes are shown as coloured bars below the sequences. The frequency of donors responding (SI ≥1.90,* P* < 0.05) is given in each bar together with the identifier for the responding donors. The core 9mer sequences of each epitope are highlighted in red and the null-mutant residues are boxed green.

T cell assays were performed by comparing deimmunised α-sarcin variants on the null-mutant α-sarcin-E96Q (this mutation was not associated with a T cell epitope, Fig. 1) background to avoid the influence of the cytotoxic effects of the protein on T cell activation. An *in vitro* co-culture of autologous CD4^+^ T cells with matured dendritic cells (DC:T cell) assay format as previously described (Holgate *et al*., 2015), including keyhole limpet haemocyanin (KLH) and humanised anti-A33 antibody controls, was used with a cohort of 20 donor PBMC to test the deimmunised α-sarcin variants at a final assay concentration of 50 µg/ml. To confirm that the deimmunised α-sarcin variants were not detrimental to DC viability, the DC harvested at Day 8, prior to irradiation and co-culture with autologous T cells, were counted and viability assessed using trypan blue (Sigma, Poole, UK) dye exclusion. Following a 7-day co-culture with autologous T cells, the cultures were pulsed with 1.0 µCi 3H-thymidine (Perkin Elmer, Buckinghamshire, UK) in 50 μl AIM-V^®^ medium and incubated for a further 6 h before harvesting and scintillation counting as above. Positive responses were defined as above where SI ≥ 1.90 (*P *< 0.05).

### *In silico* analysis of T cell epitopes

The sequences of peptides that were positive in the proliferation assay were analysed primarily using predictive iTope™ software (Perry *et al*., 2008) for HLA-DR binding. This software predicts favourable interactions between amino acid side chains of the peptide and specific binding pockets within the MHC class II binding groove. Analysis of the peptide sequences using iTope™ was performed with overlapping 9mers spanning the peptides which were tested against each of 34 MHC class II alleles. 9mers that produced a high mean binding score were identified and from the T cell proliferation data 9mers which were considered critical to T cell responses (core 9mers) were highlighted. Where no such core 9mer could be identified for peptides that were positive in the proliferation assay, further analysis was undertaken for potential binding to HLA-DQ alleles based upon HLA-DQ binding motifs (Ettinger and Kwok, 1998; Godkin *et al*., 1997; Johansen *et al*., 1996; Kwok *et al*., 1996; Sidney *et al*., 1994, 2002, 2010; van de Wal *et al*., 1997; Vartdal *et al*., 1996). Where motifs were identified, potential 9mers associated with the T cell epitopes were highlighted.

## Results

### T cell epitope mapping

A cohort of 52 donors was selected to best represent the number and frequency of HLA-DR allotypes expressed in the world population (Supplementary Fig. S1) and used to test a total of 55 overlapping peptides, which were derived from the WT α-sarcin and null-mutant (both E96Q and H137Q) amino acid sequences. Peptides were tested in the T cell assay for the presence of CD4^+^ T cell epitopes and positive T cell responses were defined by donors that produced a significant (*P *< 0.05) proliferation response with a SI ≥1.90 to any given peptide. T cell epitopes were identified by calculating the average frequency of the positive responses to all peptides in the study plus standard deviation (termed ‘background response threshold’). This was calculated to be 6.6% and equates to peptides inducing T cell proliferation (SI ≥ 1.90, *P* < 0.05) in >3 donors. All donors produced a positive T cell response against the positive control PHA in the proliferation assay, indicating that cells in the *ex vivo* cultures were functional (data not shown).

Figure 1 summarises the peptide T cell epitope map obtained from the α-sarcin sequence. Two regions contained T cell epitopes located at the N- and the C-termini of the protein. The C-terminal T cell epitope (Epitope 2) elicited slightly more frequent T cell proliferation and was considered to be more potent than the N-terminal T cell epitope (Epitope 1). For both Epitopes 1 and 2, two overlapping peptides induced proliferation responses in four or more donors and in most instances the same donors responded to both peptides. Since the core MHC class II binding 9mer will be located in at least two overlapping peptides, this result confirmed the presence of T cell epitopes in these regions. The mean magnitude of the T cell proliferation response was low for both T cell epitopes, ranging between SI 2.06 and 2.55 (Table I). Analysis of the responding donors to the N-terminal peptides containing Epitope 1 (Peptides 2 and 3) showed that there were four identical positively responding donors strongly suggesting the presence of a single T cell epitope. At least two of these donors also responded to a peptide (Peptide 4) that was not identified as containing a T cell epitope (the frequency of positive responses was below the 6.66% background response threshold). Based on these proliferation assay data, the core MHC class II binding 9mer was determined to comprise QKNPKTNKY. Assessment of this sequence using iTope™ *in silico* MHC class II binding analysis for binding against 34 HLA-DR alleles showed that this core 9mer did not bind to any of the HLA-DR alleles (data not shown). Further analysis of the core 9mer sequence revealed that it displayed motifs similar to those in HLA-DQ-restricted T cell epitopes (Ettinger and Kwok, 1998; Godkin *et al*., 1997; Johansen *et al*., 1996; Kwok *et al*., 1996; Sidney *et al*., 1994, 2002, 2010; Vartdal *et al*., 1996; van de Wal *et al*., 1997), this fact coupled with the negative result from the *in silico* HLA-DR binding analysis suggested that the T cell epitope was HLA-DQ restricted. The most likely reason Peptide 4 failed to elicit T cell proliferation responses above the background response threshold (in spite of containing the same core 9mer) is because residues lying outside the core 9mer can also play a significant role in the development of a T cell response (Arnold *et al*., 2002; Godkin *et al*., 2001; Knapp *et al*., 2009; Lovitch *et al*., 2006; Zavala-Ruiz *et al*., 2004).

**Table I.** Summary of magnitude (mean SI and range) and frequency (% donor response) of positive T cell proliferation responses against peptides containing T cell epitopes

Peptide no.	Sequence	Frequency (%)	SI range	SI mean
2	WTCLNDQKNPKTNKY	9.6	1.95–2.46	2.14
3	LNDQKNPKTNKYETK	9.6	1.91–2.52	2.15
44	VFCGIIAHTKENQGE	7.7	1.90–2.39	2.06
45	GIIAHTKENQGELKL	11.5	1.93–2.62	2.14
53	VFCGIIAQTKENQGE	7.7	1.93–2.58	2.24
54	GIIAQTKENQGELKL	9.6	1.95–5.67	2.97

The position of the p1 anchor in the potential core 9mer is shown in red.

The null mutation α-sarcin-H137Q was located within Epitope 2 and this mutation only had a modest effect on T cell proliferation compared to the equivalent WT α-sarcin peptide. The same four donors responded positively to both WT Peptide 44 and the null-mutant equivalent Peptide 53, while six and five donors (with three in common) responded positively to both WT Peptide 45 and null-mutant Peptide 54, respectively. This is strongly suggestive of a common T cell epitope that is shared between the WT and null-mutant α-sarcin-H137Q sequences. *In silico* analysis of the peptides spanning Epitope 2 (Peptides 44 and 45 for WT α-sarcin, and Peptides 53 and 54 for the null mutation α-sarcin-H137Q) identified an HLA-DR restricted binding peptide with the putative core 9mer IIAHTKENQ which bound to 20 out of a total of 34 MHC class II alleles tested by iTope™. A total of 12 alleles bound this 9mer with high affinity and with an average positive binding score of 0.6260 (on a scale of 0–1, a positive score is defined as >0.55). The null-mutant peptide IIAQTKENQ was predicted to bind to a total of 19 MHC class II alleles (in common with those that bound the WT sequence) and 12 alleles with high affinity (average binding score of 0.6196). Hence, the null-mutant α-sarcin-H137Q was predicted to generate a slightly weaker variant of Epitope 2—in agreement with the T cell epitope mapping data.

The null mutation α-sarcin-E96Q was not associated with a T cell epitope and no donors responded either to peptides containing this mutation or to the equivalent WT α-sarcin peptides. All deimmunised α-sarcin variants were, therefore, generated on the α-sarcin-E96Q background for subsequent testing as whole proteins in the *ex vivo* DC:T cell assay; however, the H137Q null mutant was used as negative control in the IVTT assay during the identification of active mutations in the T cell epitopes (both H137Q and E96Q mutants were equally inactive in the IVTT assay, data not shown).

### Epitope mutations

Based on iTope™ analysis of Peptides 44 and 45 and HLA-DQ motif analysis of Peptides 2 and 3, amino acid substitutions within the identified T cell epitopes were designed to reduce the propensity for the peptides to bind to MHC class II. Substitutions were targeted to key anchor residues of the identified core 9mer (at Positions 1, 4, 6, 7 and 9) (Hill *et al*., 1994; Jardetzky *et al*., 1990); hence, for Epitope 1, mutations were targeted to Q10 (p1), P13 (p4), T15 (p6), N16 (p7) and Y18 (p9) and for Epitope 2, mutations were targeted to I134 (p1), K139 (p6), E140 (p7) and Q142 (p9) (changes to p4 were not predicted to significantly affect MHC class II binding). Since the formation of T cell epitopes can be strongly influenced by residues adjacent to the core 9mer (e.g. p-1 and p10) (Arnold *et al*., 2002; Godkin *et al*., 2001; Lovitch *et al*., 2006; Knapp *et al*., 2009; Zavala-Ruiz *et al*., 2004), additional substitutions at Epitope 1 p-1, replacing a charged side-chain with a non-charged group, were also tested. Initially, 15 Epitope 1 and 14 Epitope 2 mutations were tested for activity individually in the IVTT assay and compared to WT α-sarcin and null-mutant α-sarcin-H137Q (Table II). The majority of mutations retained activity within 2-fold of WT α-sarcin, except I134A where a reduction in activity of 10× was observed. In a number of cases activity was significantly improved (Table II). The five most active Epitope 1 mutations were combined with the four most active Epitope 2 mutations into 20 double mutants and a further 4 mutants were constructed that contained a single Epitope 1 mutation and two Epitope 2 mutations and again tested in the IVTT assay (Table III). As before, most mutants retained activity within 2-fold of WT α-sarcin, with only two (N16K/Q142N and Y18K/Q142N) giving activity values outside this range.

**Table II.** Calculated relative IC50 values for single epitope variants tested as DNA in the IVTT assay

Mutation	Location	Relative IC50	Mutation	Location	Relative IC50
WT	–	1.00	H137Q	–	>100
D9A	Epitope 1	1.25	I134A	Epitope 2	>10
D9T	Epitope 1	0.92	K139D	Epitope 2	1.27
Q10K	Epitope 1	0.87	K139E	Epitope 2	0.88
Q10R	Epitope 1	1.23	K139G	Epitope 2	1.63
Q10A	Epitope 1	0.53	K139Q	Epitope 2	0.73
P13I	Epitope 1	0.88	K139H	Epitope 2	0.71
T15G	Epitope 1	0.62	K139N	Epitope 2	2.85
T15Q	Epitope 1	1.11	E140D	Epitope 2	0.65
T15H	Epitope 1	0.95	Q142D	Epitope 2	1.54
N16R	Epitope 1	0.86	Q142N	Epitope 2	0.96
N16K	Epitope 1	0.89	Q142T	Epitope 2	0.66
N16A	Epitope 1	0.54	Q142E	Epitope 2	1.04
Y18H	Epitope 1	0.86	Q142R	Epitope 2	0.91
Y18K	Epitope 1	0.67	Q142G	Epitope 2	0.53
Y18R	Epitope 1	0.82	–	–	–

Values were calculated by dividing the IC50 of the single epitope variant by that of WT α-sarcin assayed on the same plate.

**Table III.** Calculated relative IC50 values for double epitope variants (epitopes 1 + 2)

Mutation	Relative IC50	Mutation	Relative IC50
WT	1.00	H137Q	>100
Q10K K139D	1.16	Y18R K139D	1.05
Q10K K139E	0.98	Y18R K139E	1.37
Q10K Q142N	1.09	Y18R Q142N	1.24
N16R K139D	1.01	Q10K Q142T	0.89
N16R K139E	1.08	N16R Q142T	1.28
N16R Q142N	1.11	N16K Q142T	1.27
N16K K139D	1.62	Y18K Q142T	1.98
N16K K139E	1.79	Y18R Q142T	1.25
N16K Q142N	2.63	Q10K K139D Q142T	0.32
Y18K K139D	1.36	Q10K K139E Q142T	0.22
Y18K K139E	1.49	N16R K139D Q142T	0.27
Y18K Q142N	3.52	N16R K139E Q142T	0.22

Values were calculated by dividing the IC50 of the double epitope variant by that of WT α-sarcin assayed on the same plate.

**Fig. 2:**
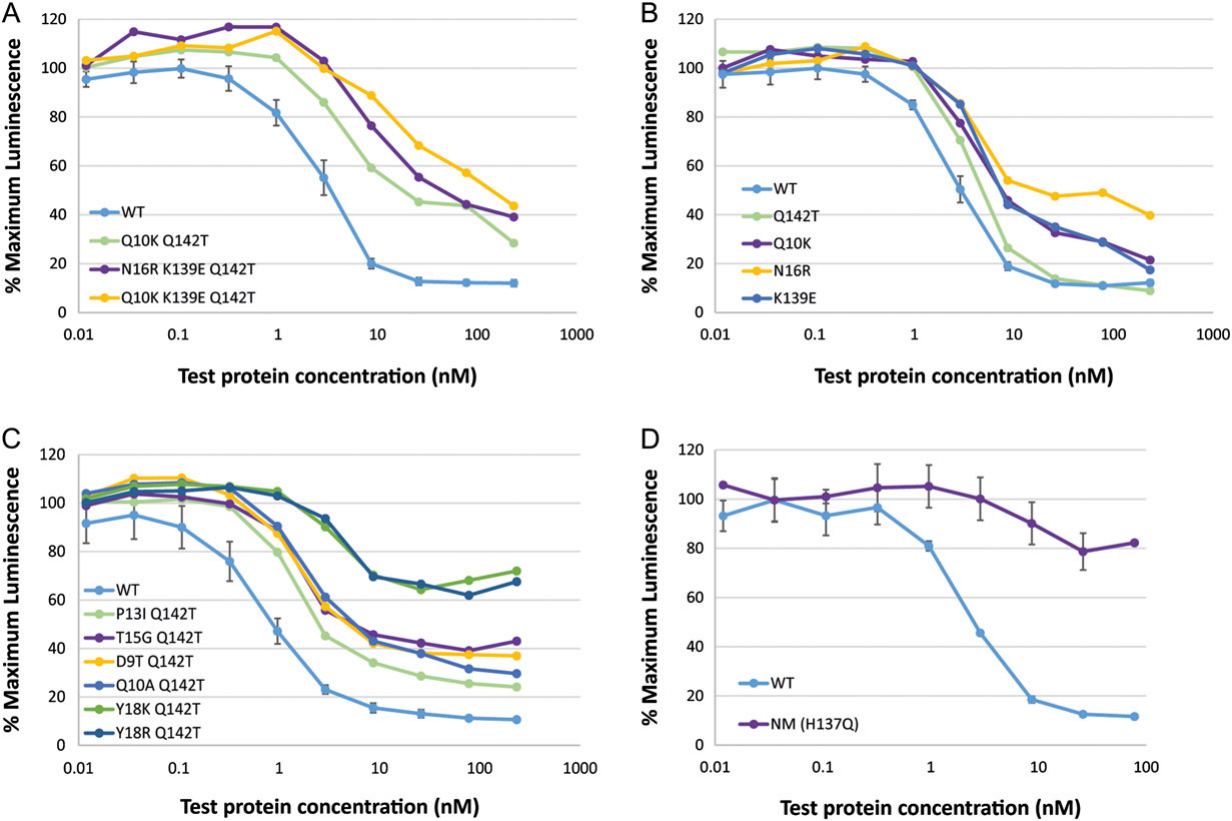
Activity of variant α-sarcin anti-Her2 scFv fusion proteins (containing a furin linker) on Her2 positive BT-474 cells. Serial dilutions of each protein were performed before combining with BT-474 cells. Following incubation at 37 °C 5% CO_2_ for 7 days, cell viability was assessed in a CellTiter-Glo luminescent cell viability assay. (**A**) Lead variants, (**B**) lead variant single mutations, (**C**) Q142T combined with alternative Epitope 1 mutations and (D) activity of null-mutant (NM) H137Q. Note: for clarity, in panels (A), (B) and (C) error bars are only shown for WT α-sarcin curve.

WT α-sarcin and null-mutant H137Q were fused to an anti-Her2 scFv separated by a linker containing a furin cleavage site, with the sequence RSKR, which was expected to enhance activity by promoting release of the toxin from the fusion protein in endosomes. The scFv fusion proteins were expressed in *E. coli* and purified to homogeneity by nickel chelate chromatography and tested for binding to Her2 positive BT-474 cells in comparison to trastuzumab alone and WT α-sarcin alone in order to confirm that fusion to trastuzumab conferred specific binding to BT-474 cells (Supplementary Fig. S2A). Cytotoxicity assays also confirmed that fusion of WT α-sarcin to trastuzumab gave a 2 log enhancement in cell killing compared to α-sarcin alone (Supplementary Fig. S2B and Supplementary Table SI) thus enhancing the natural ability of α-sarcin to penetrate and kill tumour cells (Lacadena *et al*., 2007). Therefore, the best-performing triple mutant in the IVTT assay (N16R/K139E/Q142T) together with a triple mutant containing an alternative Epitope 1 mutation that retained good activity (Q10K/K139E/Q142T) and the most active double mutant (Q10K/Q142T) were also fused to an anti-Her2 scFv separated by a furin linker, expressed, purified and tested in a cell cytotoxicity assay on BT-474 cells in comparison to the scFv fusion to WT α-sarcin (Fig. 2A and Supplementary Table SI). When fused to the anti-Her2 scFv, all three α-sarcin variants displayed at least a 5-fold reduction in activity compared to WT α-sarcin, both in terms of potency and maximum cell killing (Fig. 2A). As a result, variants containing each mutation individually were expressed as anti-Her2 scFv fusion proteins containing the furin cleavage site and tested for cytotoxicity on BT-474 cells to identify which mutation was causing the loss of activity (Fig. 2B and Supplementary Table SI). Fusion proteins containing Q10K or N16R or K139E were found to have significantly reduced activity compared to WT α-sarcin, with N16R showing the greatest drop in activity. In contrast, the activity of Q142T was comparable to WT α-sarcin (within 2-fold). Since Q142T alone was predicted to effectively reduce MHC class II binding by the C-terminal Epitope 2, a further series of six deimmunised variants was constructed based upon Q142T containing additional changes within the N-terminal Epitope 1 (D9T, Q10A, P13I, T15G, Y18K and Y18R) that had previously been shown to retain good activity in the IVTT assay (Table II). These double mutant variants were fused to anti-Her2 scFv via a furin linker and tested for activity on BT-474 cells (Fig. 2C and Supplementary Table SI). From this set of deimmunised variants, the best activity was shown by P13I/Q142T with Q10A/Q142T, D9T/Q142T and T15G/Q142T having similar but slightly lower activity; however, the potency and maximum cell killing was again reduced compared to WT α-sarcin. Deimmunised variants containing mutations at Y18 in combination with Q142T showed greatly reduced activity such that an IC_50_ value could not be calculated, although all variants were more active than the null-mutant H137Q (Fig. 2D and Supplementary Table SI).

**Fig. 3:**
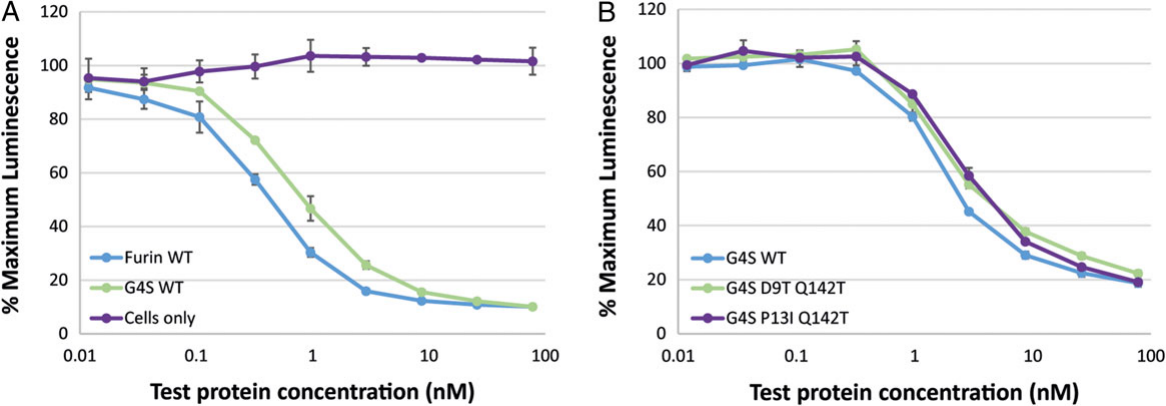
Activity of variant α-sarcin anti-Her2 scFv fusion proteins on Her2 positive BT-474 cells. Serial dilutions of each protein were performed before combining with BT-474 cells. Following incubation at 37 °C 5% CO_2_ for 7 days, cell viability was assessed in a CellTiter-Glo luminescent cell viability assay. (**A**) WT α-sarcin with either a furin or G_4_S linker, (**B**) Epitope 1 mutations D9T and P13I in combination with Q142T containing a G_4_S linker in comparison to WT α-sarcin with a G_4_S linker.

The effect of the furin linker on potency was investigated by expressing and purifying anti-Her2 scFv fusion constructs linked to WT α-sarcin either by a furin linker or a non-cleavable G_4_S linker, plus deimmunised variants D9T/Q142T and P13I/Q142T linked by a G_4_S linker. Constructs were again tested for cell killing on BT-474 cells (Fig. 3). Surprisingly, it was found that the presence of the furin linker in the WT α-sarcin constructs only had a modest beneficial effect on potency with a 2-fold improvement in toxicity compared to the G_4_S linker construct (Fig. 3A and Supplementary Table S1). Furthermore, the deimmunised variants containing the G_4_S linker possessed equivalent toxicity (within 2-fold) to the WT α-sarcin fusion also containing the G_4_S linker (Fig. 3B and Supplementary Table SI). Hence, the toxicity of the deimmunised variants with a G_4_S linker was improved compared to those containing a furin linker, both in terms of potency and maximum cell killing. Based upon these results, deimmunised variants D9T/Q142T and P13I/Q142T with the G_4_S linker were selected as lead candidates and tested in the whole protein *ex vivo* DC:T cell assay.

### Whole protein T cell assays

**Fig. 4:**
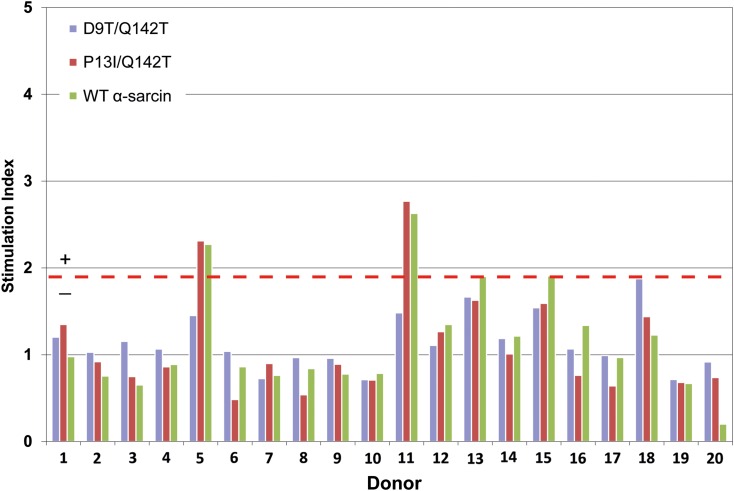
*Ex vivo* DC:T cell assay in which CD4^+^ T cell proliferation responses to deimmunised α-sarcin variants and WT α-sarcin were measured. CD4+ T cells were incubated with autologous mature DC loaded with the samples and assessed for proliferation after 7 days’ incubation. T cell responses with an SI≥1.90 (indicated by red dotted line) that were significant (*P *<0.05) using an unpaired, two sample Student's *t*-test were considered positive.

The lead deimmunised α-sarcin variants plus the WT protein were constructed and expressed containing the null mutation E96Q (Lacadena *et al*., 1999) that had previously been shown in the peptide mapping assay not to affect T cell responses and not to be associated with a T cell epitope. Mutant proteins were expressed and purified to homogeneity as described for the active deimmunised α-sarcin variants. The variants, when used at the concentrations required for the T cell assay, still retained some residual toxicity; therefore, the preferred *ex vivo* T cell assay format comprised of loading monocyte derived DC with antigen during *in vitro* maturation. Antigen was endocytosed during this antigen loading step before any remaining excess was washed from the cultures. The endocytosed antigen was processed intracellularly by the DC (during the final *in vitro* maturation step) and then autologous CD4^+^ T cells were co-cultured with the DC. This DC:T cell assay format ensured that subsequent proliferation of T cells was not influenced by the residual toxicity of the deimmunised null-mutant α-sarcin-E96Q variants. To confirm that the residual toxicity did not adversely affect DC during antigen loading/processing, the viability of DC from 10 donors was tested by trypan blue dye exclusion (8 days post-antigen loading). All cultures (including deimmunised null-mutant α-sarcin-E96Q variants, control antigens and media alone) were found to have viabilities in the range of 92–95%. T cell proliferation was induced against the assay controls KLH and humanised anti-A33 antibody (SI ≥ 1.90, *P *< 0.05) in 50% and 20% of the donor cohort, respectively. Deimmunised variant P13I/Q142T and WT α-sarcin induced positive T cell proliferation in 10% and 20% of the donor cohort, respectively, and no T cell proliferation responses were detected against deimmunised variant D9T/Q142T (Fig. 4).

## Discussion

Development of therapeutic immunotoxins that contain bacterial or plant toxins as the cell killing moiety has been hampered by undesirable immunogenicity. Such proteins, by their very nature, are immunogenic and induce immunity in humans, even after a single administration of the protein (Borthakur *et al*., 2013; Hassan *et al*., 2013, 2014; Kreitman *et al*., 2000, 2009; Schnell *et al*., 2003; Schindler *et al*., 2011). Deimmunisation through elimination of both B cell and/or T cell epitopes has been utilised in order to address the issue of immunogenicity and early progress has been made with a deimmunised version of PE38 (Mazor *et al*., 2014; Nagata and Pastan, 2009). New non-immunogenic toxin warheads need to be developed and tested in order to provide a new generation of targeted immunotoxins for the treatment of malignancies. α-Sarcin is a good candidate toxin warhead since it has been demonstrated to have potent anti-tumour activity both *in vitro* and in animal models of colorectal cancer (Carreras-Sangrà *et al*., 2012; Tomé-Amat *et al*., 2015a). In order to improve therapeutic utility, we sought to deimmunise α-sarcin by identifying T cell epitopes within the protein and then removing them by mutagenesis. Two T cell epitopes were identified in α-sarcin with core 9mer MHC class II binding sequences predicted to comprise amino acids 10–18 and 134–142. The T cell responses to peptides spanning these regions were weak, suggesting that the healthy donors had no prior exposure to α-sarcin (although phenotypic analysis of the proliferating T cells would be required to confirm this) and that α-sarcin itself is not a potent immunogen. This result is consistent with observations on the closely related fungal ribotoxin, restrictocin (Rathore and Batra, 1996).

Epitope 1 is located in a region within the N-terminal β-hairpin loop (Pérez-Cañadillas *et al*., 2000) (amino acids 7–22) that is involved in the cytotoxicity but not RNase activity of α-sarcin (53, 54 and 55). Since the deletion of this sequence leads to conformational changes within the structure, resulting in a modification of α-sarcin activity (García-Mayoral *et al*., 2004), it might be expected that mutations within Epitope 1 may affect the overall conformation of α-sarcin and could adversely affect activity. *In vitro* testing of individual Epitope 1 mutations at positions D9, Q10, P13, T15, N16 and Y18 that were predicted to remove this T cell epitope, did not have any significant effect on the overall activity of α-sarcin in the IVTT assay. None of these mutations, therefore, appeared to have any detrimental effect on the N-terminal β-hairpin and, by extension, the conformation of the deimmunised variant α-sarcin proteins. Epitope 2 lies at the end of the α-sarcin β6 sheet through to the end of loop 5 (Pérez-Cañadillas *et al*., 2000). Loop 5 makes numerous contacts with other parts of the structure via hydrophobic interactions, hydrogen bonds and salt bridges, in particular E140 has unusual backbone torsional angles. Loop 5 also makes contacts with the N-terminal β-hairpin loop principally between E140 and K11, K139 and D9, K139 and Y18, and H137 + E140 and N8 (Pérez-Cañadillas *et al*., 2000); therefore, there is a close structural association between Epitopes 1 and 2. H137 is also part of the catalytic triad (H50, E96 and H137) that is essential for α-sarcin activity (Lacadena *et al*., 1999) and, therefore, as with Epitope 1, it was possible that mutations within Epitope 2 could adversely affect the overall conformation of α-sarcin and negatively affect α-sarcin activity. Despite these complex interactions, individual mutations at positions K139, E140 and Q142 only had a modest effect on activity whereas the mutation I134A, which lies outside the loop and within the β6 sheet, resulted in markedly reduced activity, presumably by affecting the structure of the β6 sheet. Due to its key role in the stabilisation of the loop 5 structure, only one conserved mutation at E140 was tested (E140D) in order to retain the salt bridge with K11, and this mutation retained activity. Surprisingly, the activity of mutants carrying Epitope 1 mutations D9T and D9A was not significantly affected despite removing the possibility of a salt bridge with K139 within Epitope 2.

Combining the five most active Epitope 1 mutations with the four most active Epitope 2 mutations into double and triple mutant combinations resulted in a set of deimmunised variants that retained activity in the IVTT assay within 2-fold of WT α-sarcin (except N16K/Q142N and Y18K/Q142N). In particular, the triple mutant N16R/K139E/Q142T appeared to be highly active, again demonstrating that, despite these two epitopes being so closely associated and in regions important for activity, there appeared to be a significant sequence plasticity allowing retention of ribosome toxicity even when changing a positively charged residue to a negatively charged one. However, the IVTT assay only measures ribosome toxicity, whereas membrane binding and translocation, are necessary for full α-sarcin cytotoxicity. Mutational analysis of the lysine rich region (110–114) has demonstrated its interaction with phospholipids and loop 2 (Residues 53–93) is involved in membrane penetration (Castaño-Rodríguez *et al*., 2015), while the Region 116–139 has also been proposed to be involved in membrane interaction (Mancheño *et al*., 1995, 1998), with R121 specifically interacting with phospholipids (Masip *et al*., 2001). The N-terminal β-hairpin loop, and K11 in particular, has also been shown to affect membrane translocation (García-Ortega *et al*., 2001, 2002). Therefore, mutations within both Epitopes 1 and 2 have the potential to affect membrane translocation.

Two triple mutants (Q10K/K139E/Q142T and N16R/K139E/Q142T) and one double mutant (Q10K/Q142T) were fused to an anti-Her2 scFv via a furin linker, expressed, purified and tested for cytotoxicity on Her2+ BT-474 cells. In contrast to the IVTT assay, the Her2 targeted toxin variants containing the furin cleavage site were significantly less efficient at killing BT-474 cells than the Her2 scFv fusion with WT α-sarcin. Subsequent experiments showed that, with the exception of Q142T, a range of single and double mutants previously shown to have activity close to WT α-sarcin in the IVTT assay were, to varying degrees, less active when fused to the anti-Her2 targeting domain via the furin linker. The reduction in activity had two components: a reduction in potency and a reduction in maximum cell killing. These data suggested several possibilities, including: (i) the furin linker as constructed in the context of this particular fusion protein adversely affected membrane translocation, (ii) the furin linker was not efficiently cleaving, as expected; or (iii) the Her2 target and internalisation pathway influenced the trafficking, processing and escape of the fusion constructs—or all of the above could be involved.

Subsequent comparison of WT α-sarcin linked to anti-Her2 scFv either by a furin linker or a G_4_S linker showed that the cytotoxic activities on BT-474 cells of these two fusion proteins were very similar (within 2-fold) with the furin linker construct being slightly more potent. Comparison of anti-Her2 fusion proteins constructed with the most active double epitope mutants when configured with a furin linker, re-formatted with the G_4_S linker revealed that these constructs had almost the same activity as WT α-sarcin in the same configuration. Thus, the presence of a G_4_S linker appears to have enhanced the activity of the scFv-deimmunised variant fusions. These results suggest that WT and deimmunised variant α-sarcins are able to either separate from the anti-Her2 scFv in the endosomes and effectively penetrate the cell cytoplasm without requirement for a protease cleavage site or, alternatively, it is possible that the whole fusion protein with the G_4_S linker translocates to the cytoplasm and the presence of the anti-Her2 scFv does not affect ribosome binding and cytotoxicity. Similarly, other studies using a scFv to GPA33 fused with WT α-sarcin containing a G_4_S linker effectively killed cells and demonstrated anti-tumour effects *in vivo* (Carreras-Sangrà *et al*., 2012; Tomé-Amat *et al*., 2015a). It is possible that different targeting moieties (scFvs) to the same target or to different targets will traffic and process differently when fused with the new deimmunised α-sarcin variants. Therefore, optimisation of the linker may further improve the potency of these α-sarcin immunotoxins.

Based upon the collective results, deimmunised α-sarcin variants D9T/Q142T and P13I/Q142T were selected as lead candidates and tested in the whole protein *ex vivo* DC:T cell assay. WT α-sarcin was included as a control and elicited responses in 20% of the donor cohort. This suggests that α-sarcin itself has a relatively low inherent potential for immunogenicity compared to other bacterially derived immunotoxins (e.g. PE38). Moreover, this observation is consistent with the data from T cell epitope mapping, which indicated that the two T cell epitopes identified in the α-sarcin sequence were of relatively low potency, and with previous studies showing that the related fungal ribotoxin, restrictocin, was poorly immunogenic in immunised mice (Rathore and Batra, 1996). Deimmunised variant P13I/Q142T induced positive T cell responses in 10% of the donor cohort, while deimmunised variant D9T/Q142T failed to induce any responses. While the *ex vivo* T cell assays are not predictive of immunogenicity *in vivo*, we have previously shown a good correlation between these assays and clinical experience (Baker and Jones, 2007) in particular, therapeutics that have been demonstrated to be associated with low rates of ADA in the clinic give low frequencies of responses in the T cell assay; although certainty can only be demonstrated by clinical testing. Notwithstanding this caveat, variant D9T/Q142T, preferentially configured without the His tag, could be linked to any cancer-targeting binding domain to create immunotoxins with the potential for reduced risk of eliciting treatment-limiting ADA.

## Supplementary data

Supplementary data are available at *PEDS* online.

## Funding

This work was wholly funded by RCT Inc, Tucson [AZ 85711].

## Conflict of interest

K.G. is an employee of Research Corporation Technologies Inc. (RCT), who funded this work. T.J., A.H., R.H., D.K., M.F. and M.B. are employees of companies within the Abzena plc group, which were contracted by RCT to undertake the work. J.L. is an expert on α-sarcin and was an unpaid consultant to RCT for the project.

## Supplementary Material

Supplementary Data
