# Supplementary material for: A deimmunised form of the ribotoxin, α-sarcin, lacking CD4+ T cell epitopes and its use as an immunotoxin warhead
Source: Protein Eng Des Sel. 2016 Oct 22;29(11):531–40. doi: 10.1093/protein/gzw045 (PMC5081043; doi:10.1093/protein/gzw045)
Supplement: Supplementary Data [file supp_29_11_531__index.html]

A deimmunised form of the ribotoxin, α-sarcin, lacking CD4+ T cell epitopes and its use as an immunotoxin warhead — A deimmunised form of the ribotoxin, α-sarcin, lacking CD4+ T cell epitopes and its use as an immunotoxin warhead — A deimmunised form of the ribotoxin, α-sarcin, lacking CD4+ T cell epitopes and its use as an immunotoxin warhead — Supplementary Data 

# A deimmunised form of the ribotoxin, α-sarcin, lacking CD4+ T cell epitopes and its use as an immunotoxin warhead

## Supplementary Data

Supplementary Data

- Supplementary Data - pdf file
- Supplementary Data - pdf file
- Supplementary Data - pdf file
