# Supplementary material for: A deimmunised form of the ribotoxin, α-sarcin, lacking CD4+ T cell epitopes and its use as an immunotoxin warhead
Source: Protein Eng Des Sel. 2016 Oct 22;29(11):531–40. doi: 10.1093/protein/gzw045 (PMC5081043; doi:10.1093/protein/gzw045)
Supplement: Supplementary Data [file supp_gzw045_Suppl_FigureS1.pdf]

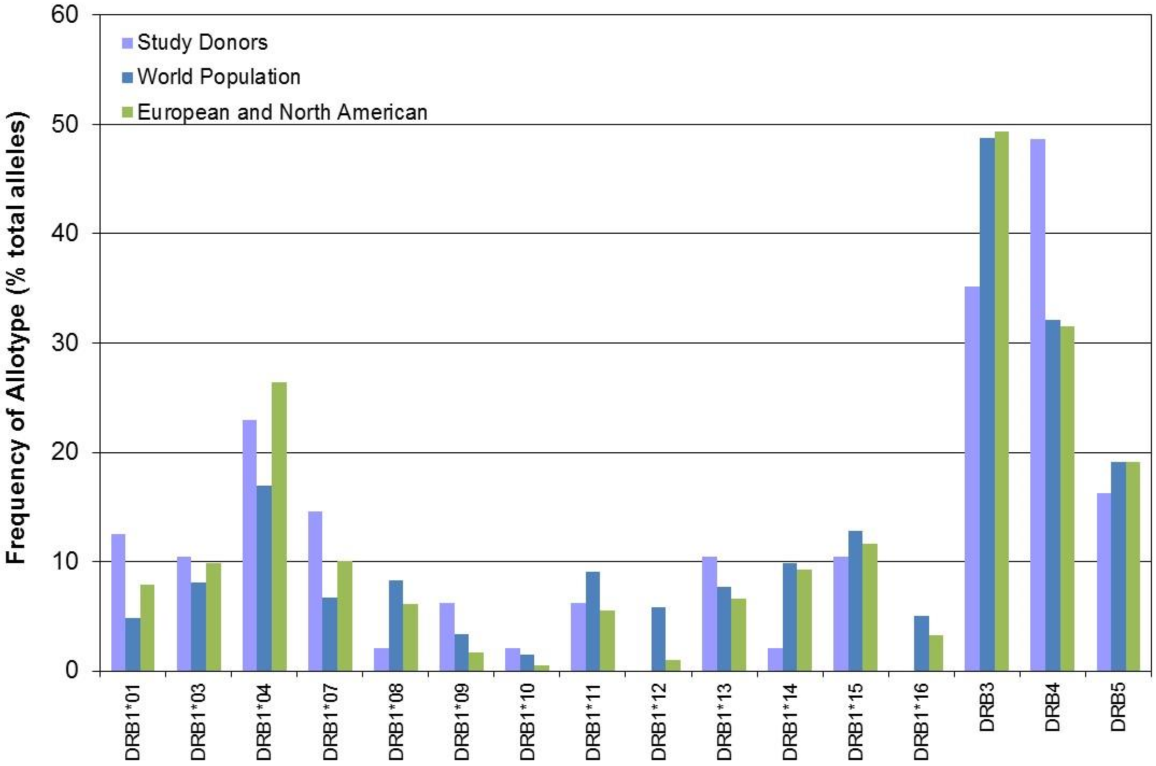


**Supplementary Figure S1**. Comparison of the frequency of HLA-DR allotypes in the current study population compared to the distribution of allotypes in the World and European and North American populations. Allotype frequency data is from: Gonzalez-Galarza et al., 2015.
