# Supplementary material for: A deimmunised form of the ribotoxin, α-sarcin, lacking CD4+ T cell epitopes and its use as an immunotoxin warhead
Source: Protein Eng Des Sel. 2016 Oct 22;29(11):531–40. doi: 10.1093/protein/gzw045 (PMC5081043; doi:10.1093/protein/gzw045)
Supplement: Supplementary Data [file supp_gzw045_Suppl_FigureS2.pdf]

A **B**

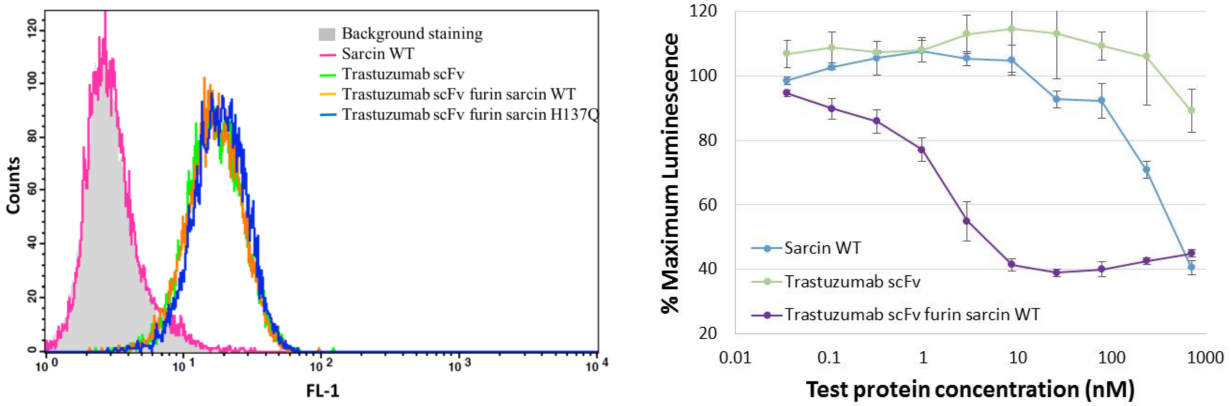

Supplementary Figure S2. Binding and activity of α-sarcin and trastuzumab scFv fusion constructs on BT474 cells: (A) Flow cytometry analysis; $3x10^5$ BT-474 cells were harvested and washed with PBS pH 7.4 before incubating with a 67 nM final concentration of test protein diluted in flow cytometry buffer (PBS pH 7.4 containing 1% BSA and 0.05% sodium azide) for 1 hour at +4°C. Cells were washed three times with the same buffer before staining with a FITC-anti-6xHis secondary antibody (Abcam, Cambridge, UK) for 1 hour at +4°C. Cells were washed three times, before binding was analysed using a FACSCalibur flow cytometer and CellQuest software (Becton Dickinson) (B) Cytotoxicity analysis; Serial dilutions of each protein were performed before combining with BT-474 cells. Following incubation at 37°C 5% CO_2 for 5 days, cell viability was assessed in a CellTiter-Glo luminescent cell viability assay.
