# Supplementary material for: A deimmunised form of the ribotoxin, α-sarcin, lacking CD4+ T cell epitopes and its use as an immunotoxin warhead
Source: Protein Eng Des Sel. 2016 Oct 22;29(11):531–40. doi: 10.1093/protein/gzw045 (PMC5081043; doi:10.1093/protein/gzw045)
Supplement: Supplementary Data [file supp_gzw045_Suppl_Tab_I.pdf]

Supplementary Table I. Summary of cellular cytotoxicity assays with all α-sarcin constructs. 'Fold Difference to Control' was calculated by dividing the IC_{50} of the test sample by the IC_{50} of the WT control assayed in the same experiment. ND indicates that an IC_{50} value could not be calculated. SD is the standard deviation of duplicate samples.

Figure	Construct			Average IC_{50} (nM)	SD	Fold Difference to Control
	Mutations	scFv?	Linker			
2A	WT	Yes	Furin	3.1	0.3	1.0
	Q10K Q142T	Yes	Furin	18.1	3.2	5.8
	N16R K139E Q142T	Yes	Furin	44.9	8.7	14.3
	Q10K K139E Q142T	Yes	Furin	133.3	19.5	42.4
2B	WT	Yes	Furin	3.0	0.2	1.0
	Q142T	Yes	Furin	4.6	0.0	1.5
	Q10K	Yes	Furin	7.7	0.9	2.5
	N16R	Yes	Furin	8.6	2.6	2.8
	K139E	Yes	Furin	12.9	1.1	4.3
2C	WT	Yes	Furin	0.9	0.1	1.0
	P13I Q142T	Yes	Furin	3.3	0.4	3.7
	T15G Q142T	Yes	Furin	6.1	1.3	6.7
	D9T Q142T	Yes	Furin	5.3	0.4	5.8
	Q10A Q142T	Yes	Furin	5.9	0.8	6.5
	Y18K Q142T	Yes	Furin	ND	ND	ND
	Y18R Q142T	Yes	Furin	ND	ND	ND
2D	WT	Yes	Furin	2.7	0.3	1.0
	NM (H137Q)	Yes	Furin	ND	ND	ND
3A	WT	Yes	Furin	0.5	0.1	1.0
	WT	Yes	G_4S	0.9	0.0	1.9
3B	WT	Yes	G_4S	3.0	0.0	1.0
	D9T Q142T	Yes	G_4S	4.5	0.2	1.5
	P13I Q142T	Yes	G_4S	4.4	0.3	1.5
Suppl 2B	WT	Yes	Furin	4.8	1.2	1.0
	WT	No	-	501.8	14.6	104.2
